# A Survey of Recent Adenoviral Respiratory Pathogens in Hong Kong Reveals Emergent and Recombinant Human Adenovirus Type 4 (HAdV-E4) Circulating in Civilian Populations

**DOI:** 10.3390/v11020129

**Published:** 2019-01-31

**Authors:** Jing Zhang, June Kang, Shoaleh Dehghan, Siddharth Sridhar, Susanna K. P. Lau, Junxian Ou, Patrick C. Y. Woo, Qiwei Zhang, Donald Seto

**Affiliations:** 1 Guangdong Provincial Key Laboratory of Tropical Disease Research, School of Public Health, Southern Medical University, Guangzhou 510515, Guangdong, China; 243562394@smu.edu.cn (J.Z.); ojx426@smu.edu.cn (J.O.); 2 Bioinformatics and Computational Biology Program, School of Systems Biology, George Mason University, Manassas, VA 20110, USA; qkang@masonlive.gmu.edu (J.K.); shoalehd@gmail.com (S.D.); 3 Chemistry Department, American University, Washington, DC 20016, USA; 4 Department of Microbiology, The University of Hong Kong, Hong Kong, China; sid8998@hku.hk (S.S.); skplau@hku.hk (S.K.P.L.); pcywoo@hku.hk (P.C.Y.W.); 5 Guangzhou Key Laboratory of Virology, Institute of Medical Microbiology, Jinan University, Guangzhou 510632, China

**Keywords:** zoonosis, host adaptation, evolution, genome recombination, respiratory pathogens, civilian populations, human adenovirus type 4

## Abstract

Human adenovirus type 4 (HAdV-E4), which is intriguingly limited to military populations, causes acute respiratory disease with demonstrated morbidity and mortality implications. This respiratory pathogen contains genome identity with chimpanzee adenoviruses, indicating zoonotic origins. A signature of these “old” HAdV-E4 is the absence of a critical replication motif, NF-I, which is found in all HAdV respiratory pathogens and most HAdVs. However, our recent survey of flu-like disease in children in Hong Kong reveals that the emergent HAdV-E4 pathogens circulating in civilian populations contain NF-I, indicating recombination and reflecting host-adaptation that enables the “new” HAdV-E4 to replicate more efficiently in human cells and foretells more potential HAdV-E4 outbreaks in immune-naïve civilian populations. Special attention should be paid by clinicians to this emergent and recombinant HAdV-E4 circulating in civilian populations.

## 1. Introduction

An increase in pediatric patients with influenza-like symptoms in Queen Mary Hospital (July to October 2014) led to a question as to whether the putatively host-adapted human adenovirus type 4 (HAdV-E4) [1] is circulating in Hong Kong. HAdV-E4 was one of the first viral respiratory pathogens to be isolated (“**R***(espiratory)***I***(nfection)*-*(number)***67**”; 1952) [2]. It is intriguingly extraordinary in several aspects, the first being that it is one of only two adenoviruses for which a vaccine was developed, thrice (1956, 1971, and 2011), at considerable effort and expense, attesting to its potency (https://www.historyofvaccines.org/content/blog/adenovirus-vaccines-reinstated-after-long-absence) [3]. Each vaccine cessation resulted in immediate reemergence as a major pathogen [4]. Second, HAdV-E4 was typically and inexplicably restricted to U.S. military populations [3,5,6], with occasional yet limited infections tallied in pathogen surveys of civilian populations [5,6,7]. Third, taxonomically, HAdV-E4 differed from other HAdVs with respect to protein chemistry properties [8] and low-resolution genomics data, including partial sequences and restriction enzyme maps [9]. Its classification, with genome sequence, as the sole HAdV comprising clade or “species” E, with 14 simian adenoviruses (SAdVs), adds to its mystique [1,10]. Origins included speculations of being the archetypical HAdV [9] or a HAdV species B and C recombinant. Genome analysis showed RI-67 was a chimpanzee adenovirus (ChAdV) [1,10], with a genome identity of 89.8% and 89.2% to SAdV-E26 and SAdV-E25, respectively. These are much higher than two respiratory pathogens HAdV-B7 at 73.7% and HAdV-C1 at 65.7%, with ocular pathogen HAdV-D9 at 70.4%.

The zoonotic threat potential of adenoviruses is recognized [11], and HAdV-E4 represents the first example. Notably, both the HAdV-E4 prototype and contemporaneous “vaccine” isolates contain a genome signature that is unique to ChAdVs and other SAdVs, i.e., the absence of the Nuclear Factor I (NF-I) binding site that is conserved among the three replication motifs embedded in nearly all HAdV inverted terminal repeats (ITRs) [1]. NF-I is a host transcription factor that binds to nt 23–36 of the HAdV-2 origin of replication [12,13,14,15,16,17,18,19,20] and is recruited by the human adenoviral replication complex [12,17]. It is firmly established as essential through in vitro and in vivo studies (see [18,19] and *qtd. in*), including replication reconstitution assays, as necessary for efficient adenoviral replication in human cells (see [20] and *qtd. in*). Recent isolates are recombinants containing this HAdV replication motif [1], presumably permitting an expansion of the virus range into the immune-naïve populations [5,6,7,21,22,23], and should be noted as a molecular evolution example of a post-zoonotic, host-adaptation of a “novel” and “emergent” human pathogen.

## 2. Results and Discussion

**Figure 1:**
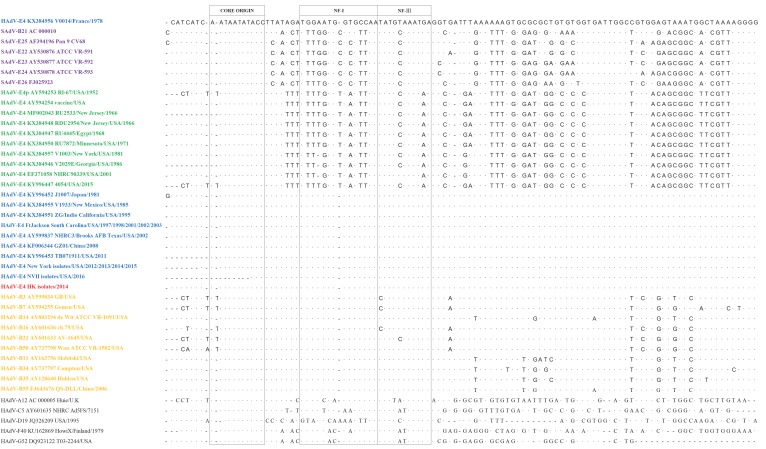
Human adenovirus Inverted Terminal Repeats (116–209 bases) embed replication protein-binding motifs within 88 bases: “Core origin”, NF-I (Nuclear Factor I), and NF-III. Host transcription factors NF-I and NF-III enable adenovirus replication in human cells. The first recorded host-adapted HAdV-E4 (Human adenovirus type 4) ITR (inverted terminal repeats) is arrayed along the top (V0014/France/1978), contrasted against differences in motifs from SAdVs (simian adenoviruses) (lavender) and prototype-like HAdV-E4 (green). Dots indicate conserved bases. Recent host-adapted HAdV-E4 isolates (blue), including five isolates from Ft. Jackson, spanning six years, and 10 recent Hong Kong isolates (red) contain these motifs and are nearly indistinguishable from motifs from HAdV respiratory pathogens (yellow). The majority of HAdVs possess these motifs (black), with the most divergent being HAdV-D19.

As noted in the literature, the ITR contains critical conserved DNA replication motifs that include the human transcription factor binding site, NF-I, which is shown to enhance and optimize HAdV replication and growth [2,12,13,14,15,16,17,18,19,20]. All of the 10 Hong Kong isolates (red) possess this host-adapted ITR, i.e., they have the NF-I binding site that is found in all human respiratory adenoviruses (yellow) (Figure 1). However, this motif is missing in SAdV ITRs (purple) [18], as well as the “old” HAdV-E4 strains (green), e.g., prototype (1952) and “vaccine” (1962) strains, i.e., they have only the core origin and the NF-III motif [1]. The absence of the NF-I motif likely plays a role in limiting the circulation of both SAdVs and prototype HAdV-E4 in human populations, as its acquisition presumably provides efficient and enhanced replication in human cells, i.e., allowed HAdV-E4 to adapt to the new host. This may be the “tipping point” that allows HAdV-E4 entry into the general population that is immunologically-naïve to its epsilon antigen, as HAdV-E4 does not normally circulate outside the U.S. military populations [3,5,6,7]. This is consistent with reports documenting recombination as an evolution mechanism in the genesis of emergent HAdV pathogens [24], illustrated by HAdV-D53 [25] and HAdV-B55 [24]. HAdV-B55 is a recent major respiratory pathogen in China, after years of quiescence, by virtue of its introduction into immunologically-naïve populations, i.e., without antibodies to its HAdV-B11-like epsilon antigen. Another example is the reemergence of a long-dormant respiratory pathogen HAdV-B14 into an immunologically-naïve population [26].

Consistent with this report, recombination has been reported in SAdVs [27,28] and cross-species transmissions between humans and non-human simians have been reported [28,29,30,31,32,33,34], including TMAdV across “two passages” of human hosts, with clinical manifestations [34], as well as seroprevalence of adenoviruses across species [29,32,33,34]. These support the hypothesis and data suggesting HAdV-E4 was of zoonotic origins [1,10].

**Figure 2:**
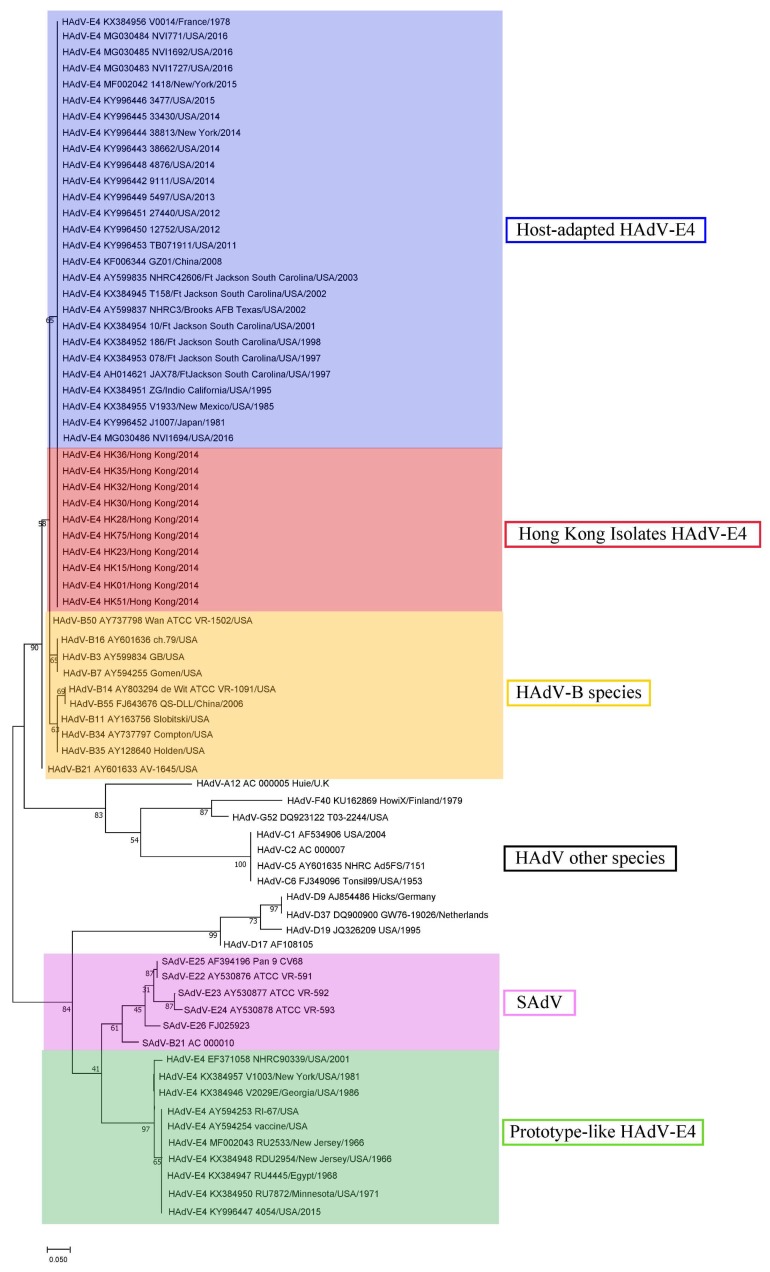
Phylogeny of select HAdV and SAdV ITRs. Recent host-adapted HAdV-E4, including Hong Kong isolates, subclade with HAdVs. Prototype-like HAdV-E4 isolates subclade with SAdVs. Inverted terminal repeats (ca., 116 bases) were aligned using Molecular Evolutionary Genetics Analysis (MEGA) 7.0.26 software (https://www.megasoftware.net), and phylogenetic trees were constructed with a maximum-likelihood method incorporating 1000 bootstrap replications.

Since sequences diverge considerably downstream of the NF-III motif, a phylogenetic analysis of the full ITR (116–209 bases) was completed (Figure 2). Recent host-adapted “new” HAdV-E4 isolates, including Hong Kong isolates, subclade with human respiratory adenoviruses of species B as well as with other HAdVs. Prototype-like HAdV-E4 isolates subclade with SAdVs.

Comparison of HAdV-E4 genomes spanning 65 years reveals highly conserved sequences. For example, the two earliest genomes, isolated within 10 years, are 99.9% identical to each other, differing by 18 mismatches and two insertions (one one-base and one three-base) [35]. One striking difference is a recombination resulting in recent HAdV-E4 genomes acquiring a well-characterized replication-enhancing NF-I motif that distinguishes these isolates from the 1952 prototype and a contemporaneous 1965 co-circulating strain. Although the acquisition of this critical replication motif alone may explain the potentially wider distribution of HAdV-E4, the pair-wise sequence analyses also reveal additional potentially important sequence differences in the E3 gene region [36]. Proteins encoded in the E3 region are involved in interactions with the host immune response and evasion and represent the most variable region between HAdV types [37,38,39]. This report highlights the need to re-explore the understudied E3 genes and their critical roles in the human immune response upon HAdV infection.

However, whether in concert with E3 gene changes or alone, the NF-I acquisition, given “Occam’s Razor”, may be the critical evolutionary modification and “tipping point” that is necessary for a wider distribution of HAdV-E4 in immune-naïve civilian populations in light of the extensive literature reported for HAdV replication requirements [12,13,14,15,16,17,18,19,20]. This host adaptation may be significant as HAdV-E4 is a highly contagious pathogen with demonstrated morbidity and mortality implications and has now been reported in global civilian populations [6,7,23]. During the preparation of this report, to emphasize further the potential of HAdV-E4 emergence in the global general population, seroepidemiological data identifying HAdV-E4 infections in China and Sierra Leone (Africa) were published [40]. Explicitly, the authors note “An apparent increase in the frequency of human adenovirus type 4 (HAdV-4) infections among general populations has been observed over the past 10 years” [40].

## 3. Materials and Methods

### 3.1. Clinical Specimens and Virus Culture

Nasopharyngeal swab specimens were collected from both outpatients and inpatients who presented with flu-like symptoms, and are archived at Queen Mary Hospital (Hong Kong). Adenoviruses were detected by PCR and were identified further by molecular typing using partial sequence data from the hexon and fiber genes, as previously reported [41]. Then, 10 virus strains that were identified as type 4 were cultured in human lung cell lines (A549), and genomic DNA was extracted using the Viral DNA Extraction Kit from Omega Bio-Tek, Inc. (Norcross, GA, USA), as previously noted [41].

### 3.2. DNA Sequencing and Bioinformatic Analyses

ITRs were sequenced using genomic DNA as a template, with either a 5’-primer “Ad4-ITR-L” (5’-AACTCTTCTCGCTGGCACTCAA-3’) or a 3’-primer “Ad4-ITR-R” (5’-CCGCCCCTAACAGTCGCC-3’). These Sanger chemistry-derived ITR sequence data generated with an ABI3730 DNA sequencer were aligned and parsed for sequence motifs. Phylogenetic analysis was undertaken with Molecular Evolutionary Genetics Analysis software (MEGA) 7.0.26 (https://www.megasoftware.net), for which FASTA files were inputted for sequence alignments utilizing a Maximum Composite Likelihood method to generate neighbor-joining, bootstrapped phylogenetic trees with 1000 iterations [1,42]. All other MEGA program parameters were set by default. Bootstrap values above 80 are considered robust. For pair-wise genome comparisons, zPicture (http://zpicture.dcode.org/) was used to provide detailed global insights.

### 3.3. Sequence Data

All sequences are available in GenBank from which whole genome data were retrieved and aligned, and ITR sequences extracted. This resulted in a data set of ITR sequences ranging from 116 to 209 bases, as lengths were variable among the types. A subset of sequences spanning the first 1–66 bases that included the conserved HAdV viral replication sequence motifs were selected for detailed analysis.

Genome sequence data were accessed from GenBank. HAdV-E4 genomes include RI-67 or “prototype” (Ft Leonard Wood, Missouri; 1952; AY594253); CL68578 or “vaccine” (Camp Lejeune, North Carolina; 1965; AY487947); V0014 (France; 1978; KX384956); JAX78 (Ft Jackson, South Carolina; 1997; KX384953); and NHRC3 (Brooks AFB, Texas; 2002; AY599837); NVI771 (USA; 2016; MG030484); NVI692 (USA; 2016; MG030485); NVI727 (USA; 2016; MG030483); 1418 (New York; 2015; MF002042); 3477 (USA; 2015; KY996446); 33430 (USA; 2014; KY996445); 38813 (New York; 2014; KY996444); 38662 (USA; 2014; KY996443); 4876 (USA; 2014; KY996448); 9111 (USA; 2014; KY996442); 5497 (USA; 2013; KY996449); 27440 (USA; 2012; KY996451); 12752 (USA; 2012; KY996450); TB071911 (USA; 2011; KY996453); GZ01 (China; 2008; KF006344); NHRC42606 (Ft Jackson, South Carolina; 2003; AY599835); T158 (Ft Jackson, South Carolina; 2002; KX384945); 10 (Ft Jackson, South Carolina; 2001; KX384954); 186 (Ft Jackson, South Carolina; 1998; KX384952); 078 (Ft Jackson, South Carolina; 1997; KX384953); ZG (Indio, California; 1995; KX384951); V1933 (New Mexico; 1985; KX384955); J1007; Japan; 1981; KY996452); NVI1694 (USA; 2016; MG030486); NHRC90339 (USCG Training Ctr (Cape May), Delaware; EF371058); V1003 (New York; 1981; KX384957); V2029E (Georgia; 1986; KX384946); RU2533 (New Jersey; 1966; MF002043); RDU2954 (New Jersey; 1966; KX384948); RU4445 (Egypt; 1968; KX384947); RU7872 (Minnesota; 1971; KX384950); and 4054 (USA; 2015; KY996447). Additional representative HAdV genomes include HAdV-B50 (Wan; AY737798); HAdV-B16 (ch.79; AY601636); HAdV-B3 (GB; AY599834); HAdV-B7 (Gomen; AY594255); HAdV-B14 (de Wit; AY803294); HAdV-B55 (QS-DL; FJ643676); HAdV-B11 (Slobitski; AY163756); HAdV-B34 (Compton; AY737797); HAdV-B35 (Holden; AY128640); HAdV-B21 (AV-1645; AY601633); HAdV-A12 (Huie; AC_000005); HAdV-F40 (HowiX; FH162869); HAdV-G52 (T03-2244; DQ923122); HAdV-C1 (AF534906); HAdV-C2 (AC_000007); HAdV-C5 (7151; AY601635); HAdV-C6 (Tonsil 99; FJ349096); HAdV-D9 (Hicks; AJ854486); HAdV-D37 (GW76-19026; DQ900900); HAdV-D19 (JQ326209); and HAdV-D17 (AF108105). Simian adenoviral genomes include SAdV-B21 (AC_000010); SAdV-E22 (AY530876); SAdV-E23 (AY530877); SAdV-E24 (AY530878); SAdV-E25 (AF394196); and SAdV-E26 (FJ025923). The ITR sequences of Hong Kong HAdV-E4 isolates are archived in GenBank (KY062614–KY062623).
